# Human Chorionic Plate-Derived Mesenchymal Stem Cells Restore Hepatic Lipid Metabolism in a Rat Model of Bile Duct Ligation

**DOI:** 10.1155/2017/5180579

**Published:** 2017-11-09

**Authors:** Yun Bin Lee, Jong Ho Choi, Eun Nam Kim, Jin Seok, Hyun-Jung Lee, Jung-Hwan Yoon, Gi Jin Kim

**Affiliations:** ^1^Department of Internal Medicine, CHA Bundang Medical Center, CHA University, Seongnam, Republic of Korea; ^2^Department of Biomedical Science, CHA University, Seongnam, Republic of Korea; ^3^Department of Dermatology, The Feinberg School of Medicine, Northwestern University, Chicago, IL, USA; ^4^Clinical Research Center, CHA Bundang Medical Center, CHA University, Seongnam, Republic of Korea; ^5^Department of Internal Medicine and Liver Research Institute, Seoul National University College of Medicine, Seoul, Republic of Korea

## Abstract

In cholestatic liver diseases, impaired bile excretion disrupts lipid homeostasis. We investigated changes of lipid metabolism, including mitochondrial *β*-oxidation, in a rat model of bile duct ligation (BDL) in which chorionic plate-derived mesenchymal stem cells (CP-MSCs) were transplanted. Serum cholesterol level, which was elevated after BDL, was significantly decreased following CP-MSC transplantation. The expression levels of genes involved in intracellular lipid uptake, including long-chain fatty acyl-CoA synthetases and fatty acid transport proteins, were decreased in rats after BDL; however, they were not significantly changed by subsequent CP-MSC transplantation. Carnitine palmitoyltransferase 1A (CPT1A), a rate-limiting enzyme in mitochondrial *β*-oxidation, was upregulated after BDL and then was downregulated after CP-MSC transplantation. CPT1A expression was changed via microRNA-33—a posttranscriptional regulator of CPT1A—in a peroxisome proliferator-activated receptor *α*-independent manner. Cellular adenosine triphosphate production—an indicator of mitochondrial function—was reduced after BDL and was restored by CP-MSC transplantation. Expression levels of heme oxygenases also were significantly affected following BDL and CP-MSC transplantation. Lipid metabolism is altered in response to chronic cholestatic liver injury and can be restored by CP-MSC transplantation. Our study findings support the therapeutic potential of CP-MSCs in cholestatic liver diseases and help in understanding the fundamental mechanisms by which CP-MSCs affect energy metabolism.

## 1. Introduction

Cholestatic liver injury, which is caused by accumulation of bile acids and lipids, comprises a wide spectrum ranging from acute transient hepatitis to cirrhosis with portal hypertension [1–3]. The liver controls central processes of lipid metabolism including fatty acid synthesis, mitochondrial *β*-oxidation, and phospholipid transport. Impaired bile excretion, caused by biliary obstruction or liver damage, disrupts cholesterol and phospholipid metabolism [4]. In a rat model of bile duct ligation (BDL), serum levels of very low-density lipoprotein cholesterol and low-density lipoprotein (LDL) cholesterol are drastically elevated, whereas hepatic lipid concentrations are unchanged [5]. However, alterations in mitochondrial function in chronic cholestatic liver diseases have not been elucidated.

Mesenchymal stem cells (MSCs) are multipotent adult stem cells that can differentiate into various cell types of the three germ layers (i.e., the ectoderm, mesoderm, and endoderm) [6]. The human placenta is an abundant source of MSCs. Placenta-derived MSCs (PD-MSCs) which originate from the fetus possess great potential for self-renewal, proliferation, and differentiation [7, 8]. We previously found that full-term placenta harbors several types of PD-MSCs, including chorionic plate-derived MSCs (CP-MSCs), chorionic villus-derived MSCs, and Wharton's jelly-derived MSCs [9]. CP-MSCs are highly capable of differentiating into various lineage cells, including hepatocytes. Moreover, CP-MSCs have been demonstrated to have anti-inflammatory, antifibrotic, and proregenerative properties in the damaged liver [10, 11].

We therefore used a BDL rat model of chronic cholestatic liver injury to clarify the alterations in hepatic lipid homeostasis—focusing on mitochondrial dysfunction—and the impact of CP-MSC transplantation restoring the alterations in hepatic lipid metabolism.

## 2. Materials and Methods

### 2.1. Cell Culture

Collection of placenta samples for research purposes was approved by the Institutional Review Board of CHA Gangnam Medical Center, Seoul, Korea (IRB 07-18). All participants provided written informed consent prior to sample collection. Placentas were obtained from women who were free of any medical, obstetrical, or surgical complications and who delivered at term (38 ± 2 gestational weeks). CP-MSCs were isolated as described previously [10] and were cultured in Dulbecco's modified Eagle medium/Ham's F-12 medium (DMEM/F12; Sigma-Aldrich, St. Louis, MO, USA) supplemented with 10% fetal bovine serum (FBS; Sigma-Aldrich), 1% penicillin/streptomycin (Sigma-Aldrich), 1 *μ*g/mL heparin (Sigma-Aldrich), and 25 ng/mL human fibroblast growth factor-4 (hFGF-4; Peprotech Inc., Rocky Hill, NJ, USA) at 37°C in a 5% CO_2_ incubator containing 20% O_2_.

### 2.2. BDL Rat Model and Transplantation of CP-MSCs

Male 7-week-old Sprague-Dawley rats (Orient Bio Inc., Seongnam, Korea) were maintained in an air-conditioned animal facility. The common bile duct was ligated under general anesthesia with Avertin (2,2,2-tribromoethanol, Sigma-Aldrich) as described previously [12, 13]. One week after BDL, CP-MSCs (2 × 10^6^ cells, 8–10 passages) were injected intravenously via tail vein in the transplanted group. The CP-MSC number was determined based on the previous dose-determining experiments [10, 14]. Liver tissues and blood samples were collected 1, 2, 3, and 5 weeks posttransplantation in the transplanted group and 1, 2, 3, and 5 weeks post-BDL in the nontransplanted group. The experimental protocols were approved by the Institutional Animal Care and Use Committee of CHA University, Seongnam, Korea (IACUC-140009).

### 2.3. Histological Analysis

Liver tissue samples were fixed in 10% formalin, embedded in paraffin, and sectioned at 5 *μ*m thickness. Sections then were stained with hematoxylin and eosin and observed under light microscopy at 200x magnification (Axioskop2, Carl Zeiss Micro-Imaging, Oberkochen, Germany).

### 2.4. Immunofluorescence Staining

To analyze the expression of carnitine palmitoyltransferase 1A (CPT1A) in liver tissues, 6 *μ*m thick cryostat sections were incubated with protein blocking solution (Dako, Glostrup, Denmark) for 40 minutes at room temperature. Then, a mouse anti-CPT1A antibody (1 : 100, Abcam, Cambridge, MA, USA) was treated, and sections were incubated at 4°C overnight. After washing with phosphate-buffered saline (PBS), samples were incubated with an Alexa 488-conjugated secondary antibody (1 : 150, Invitrogen, Carlsbad, CA, USA) for 1 hour at room temperature. Sections then were stained with 4′,6-diamidino-2-phenylindole (DAPI) for nuclear counterstaining and were observed under fluorescence microscopy at 400x magnification (Nikon, Tokyo, Minato, Japan).

### 2.5. Blood Chemistry

The serum concentrations of total cholesterol, high-density lipoprotein (HDL) cholesterol, LDL cholesterol, triglyceride, albumin, total bilirubin, alkaline phosphatase (ALP), aspartate transaminase, alanine transaminase, and C-reactive protein (CRP) were measured enzymatically by an automated analyzer (Hitachi 747, Hitachi, Tokyo, Japan).

### 2.6. Fatty Acyl-CoA Synthetase Activity Assay

Long-chain fatty acyl-CoA synthetase (ACSL) activity was assessed by the enzyme-linked immunosorbant assay (ELISA). Liver tissues were homogenized in cold PBS with a glass homogenizer on ice. ACSL activity was measured using a Rat Fatty acyl-CoA synthetase ELISA Kit (MyBioSource, San Diego, CA, USA) in strict accordance with the manufacturer's instructions and detected using a microplate reader (BioTek, Winooski, VT, USA) at 450 nm.

### 2.7. Quantitative Real-Time Polymerase Chain Reaction

Rat liver tissues were homogenized and lysed, and total RNA was isolated with the TRIzol reagent (Invitrogen). Reverse transcription was performed with 500 ng of total RNA and Superscript III reverse transcriptase (Invitrogen). Real-time polymerase chain reaction (PCR) was performed with SYBR Green PCR Master Mix (Applied Biosystems, Foster City, CA, USA). The cDNA subsequently was amplified by PCR using the following thermal conditions: 5 minutes at 95°C, 40 cycles of 95°C for 5 seconds, and 60°C for 30 seconds. The sequences of the primers are listed in Table 1. GAPDH or *β*-actin was used as an internal control for normalization.

### 2.8. Isolation and Quantification of MicroRNA-33

Total RNA was isolated with the TRIzol reagent (Invitrogen) and reverse-transcribed with a Mir-X miRNA First-Strand Synthesis Kit (Clontech, Mountain View, CA). Then, real-time PCR for microRNA-33 (miR-33) was performed using the following primer: 5′-GTG CAT TGT AGT TGC ATT GCA-3′ (forward). The expression of miR-33 was normalized to U6 snRNA expression.

### 2.9. Western Blot Analysis

Liver tissues were homogenized and lysed on ice with RIPA buffer containing protease inhibitor cocktail (Roche, Branchburg, NJ, USA) and a phosphatase inhibitor (Sigma-Aldrich). Protein lysates were separated by 8% to 15% sodium dodecyl sulfate polyacrylamide gel electrophoresis (SDS-PAGE), transferred to polyvinylidene difluoride membranes (Bio-Rad Laboratories, Hercules, CA, USA), and then blocked in blocking buffer (0.1% Tween20 and 8% bovine serum albumin [BSA] in Tris-buffered saline [TBS]) for 1 hour. Membranes subsequently were incubated with mouse anti-CPT1A (1 : 1000, Abcam), rabbit anti-peroxisome proliferator-activated receptor *α* (PPAR*α*) (1 : 1000, Abcam), and rabbit anti-GAPDH (1 : 3000, Santa Cruz Biotechnology, Santa Cruz, CA, USA) antibodies at 4°C overnight. After the reaction, membranes were treated with a horseradish peroxidase- (HRP-) conjugated secondary antibody (anti-rabbit IgG [1 : 25000, Bio-Rad Laboratories] or anti-mouse IgG antibody [1 : 25000, Bio-Rad Laboratories]) for 1 hour at room temperature. The bands were detected using an enhanced chemiluminescence reagent (Bio-Rad Laboratories).

### 2.10. Adenosine Triphosphate Assay

Adenosine triphosphate (ATP) concentrations of homogenized liver tissue samples were measured using an ATP assay kit (Abcam), according to the manufacturer's instructions, and were assessed using a microplate reader (BioTek) at 570 nm.

### 2.11. Statistical Analysis

All experiments were conducted in duplicate or triplicate. Data are expressed as mean ± standard deviation. Student's *t*-tests were performed for groupwise comparisons, and *P* < 0.05 was considered statistically significant. Statistical analyses were performed using PASW version 22.0 (SPSS Inc., Chicago, IL, USA).

## 3. Results

### 3.1. CP-MSC Transplantation Ameliorates Inflammation in the BDL Rat Liver

**Figure 1:**
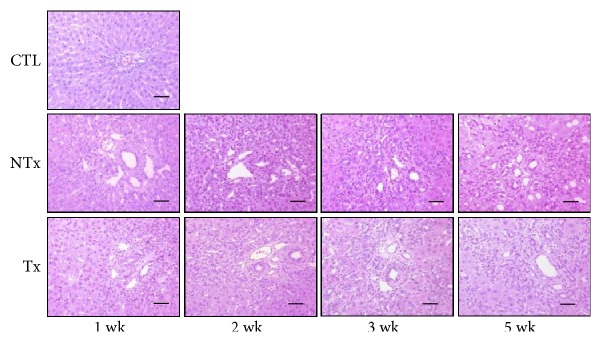
Inflammatory response induced by chronic cholestasis and the effect of CP-MSC transplantation. Histological analysis with hematoxylin and eosin staining (scale bar = 50 *μ*m; original magnification, ×200). CTL: control group; NTx: nontransplanted group; Tx: transplanted group.

To assess the effect of transplantation of CP-MSCs on cholestatic liver injury, BDL rats were divided into 2 groups: rats in the transplanted group were injected with CP-MSCs, and rats in the nontransplanted group were injected with the culture medium. As shown in Figure 1, we observed the infiltration of inflammatory cells around bile ducts and bile duct proliferation in portal areas in both nontransplanted and transplanted groups 1 week after BDL. Two weeks after BDL, portal areas were expanded as a result of extensive bile duct proliferation and concentric periductal fibrosis, and disorganization of normal lobular structures was observed in the nontransplanted group. Bile duct proliferation was less prominent, and the lobular pattern was preserved in the transplanted group compared to the nontransplanted group (Figure 1). Hepatic steatosis was not observed in the control, nontransplanted, or transplanted groups.

### 3.2. CP-MSC Transplantation Attenuates BDL-Induced Hypercholesterolemia but Does Not Affect Fatty Acid Uptake

**Figure 2:**
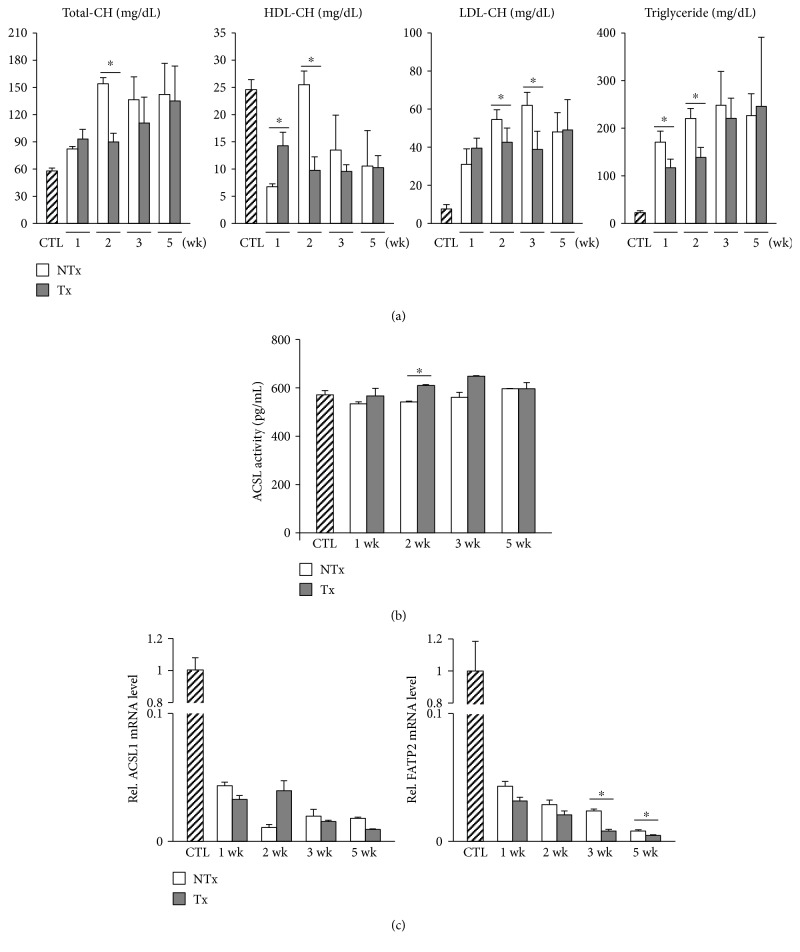
Changes in serum lipid profiles and expression levels of genes associated with intracellular uptake of fatty acids after BDL and/or CP-MSC transplantation. (a) Serum levels of total cholesterol, HDL cholesterol, LDL cholesterol, and triglyceride. (b) Activities of ACSL, as measured by ELISA. (c) mRNA expression levels of ACSL1 (left) and FATP2 (right). *β*-Actin was used as an internal control for normalization. Data are expressed as a fold change related to the control group. ^∗^*P* < 0.05 (compared to the nontransplanted group). CTL: control group; NTx: nontransplanted group; Tx: transplanted group.

Obstruction of bile excretion induced by BDL results in overflow of biliary phospholipids in the circulation [4]. Therefore, we explored the effect of transplantation of CP-MSCs on cholesterol metabolism by measuring the cholesterol concentrations in serum. Total cholesterol was markedly elevated in the nontransplanted group 2 weeks after BDL compared to the control group, whereas it was significantly reduced in the transplanted group compared to the nontransplanted group (*P* < 0.05; Figure 2(a)). Results similar to those for total cholesterol were found for the concentrations of serum LDL cholesterol and triglyceride (Figure 2(a)). Increases in serum levels of total bilirubin, ALP, and CRP were shown to be attenuated after transplantation of CP-MSCs (*P* < 0.05; Figure S1 in Supplementary Material available online at https://doi.org/10.1155/2017/5180579).

Because hypercholesterolemia is induced by chronic cholestasis, we hypothesized that fatty acid uptake into hepatocytes may be altered in BDL rats. ACSLs and fatty acid transport proteins (FATPs) are thought to be essential for the intracellular uptake and transport of fatty acids [15, 16]. Therefore, we determined the activity of ACSLs and the expression levels of ACSLs and FATPs in rat liver tissues. ACSL activity—measured by ELISA—was increased significantly in the transplanted group compared to the nontransplanted group (*P* < 0.05; Figure 2(b)). The expression levels of ACSL1, which is highly expressed in the normal liver [17], were decreased in BDL rats; however, they were not increased significantly by CP-MSC transplantation (Figure 2(c)). The expression levels of ACSL4 and ACSL5, which are located in rat liver peroxisomes and mitochondria, respectively [18], declined drastically after BDL and were not restored by CP-MSC transplantation (Figure S2). The expression levels of FATP2 and FATP5, which are expressed in hepatocytes [19, 20], were decreased in BDL rats and were not increased significantly by CP-MSC transplantation (Figures 2(c) and S3). Collectively, these findings indicate that cholestasis and hypercholesterolemia induced by BDL are ameliorated by CP-MSC transplantation. However, transplantation of CP-MSCs does not appear to restore processes of fatty acid import into hepatocytes.

### 3.3. CPT1A Expression Is Changed via MiR-33 in BDL Rats

**Figure 3:**
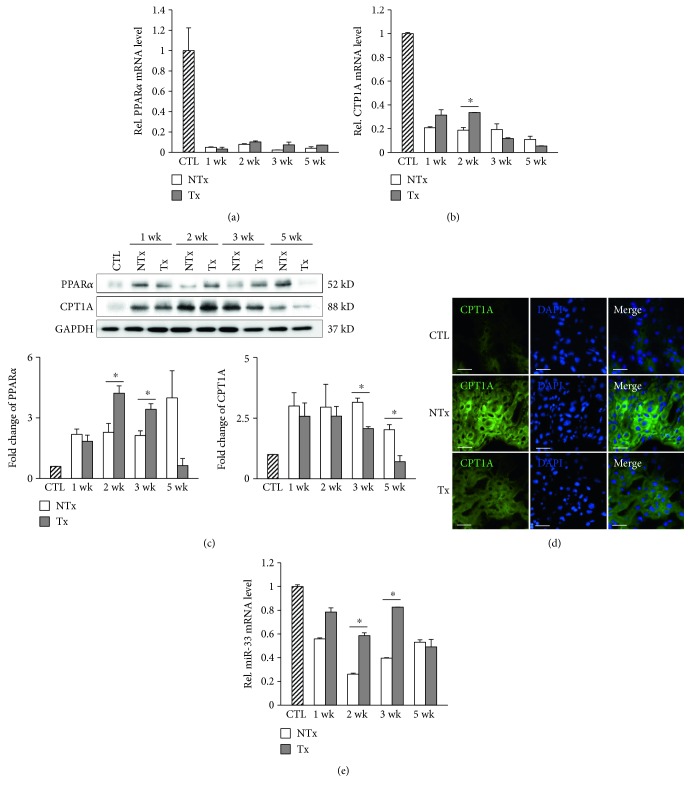
Expression of genes associated with fatty acid oxidation after BDL and/or CP-MSC transplantation. mRNA expression levels of PPAR*α* (a) and CPT1A (b) by real-time PCR. *β*-Actin was used as an internal control for normalization. Data are expressed as a fold change related to the control group. (c) Protein expression levels of PPAR*α* and CPT1A. GAPDH was used as a loading control, and quantification by densitometry of Western blots was normalized to GAPDH. Data are expressed as a fold change related to the control group. (d) Analysis of CPT1A expression with immunofluorescence staining (scale bar = 200 *μ*m; original magnification, ×400). Liver tissues, which were collected at 3 weeks posttransplantation in the transplanted group and post-BDL in the nontransplanted group, were used in immunofluorescence staining. (e) mRNA expression levels of miR-33. U6 snRNA was used as an internal control for normalization. ^∗^*P* < 0.05 (compared to the nontransplanted group). CTL: control group; NTx: nontransplanted group; Tx: transplanted group.

CPT1A is a rate-limiting enzyme located in the mitochondrial outer membrane that catalyzes *β*-oxidation of free fatty acid [21]. PPAR*α* regulates mitochondrial and peroxisomal fatty acid oxidation by controlling downstream genes, such as CPT1A [22]. We investigated whether the expression of genes associated with fatty acid oxidation is altered in BDL rats and restored by transplantation of CP-MSCs. The mRNA levels of PPAR*α* and CPT1A were remarkably decreased after BDL (Figures 3(a) and 3(b)). PPAR*α* mRNA levels were similar in the nontransplanted and transplanted groups (Figure 3(a)); however, CPT1A mRNA expression was significantly augmented 2 weeks after CP-MSC transplantation (*P* < 0.05; Figure 3(b)). On the contrary, the increased protein expression levels of CPT1A by BDL were reinstated to near-control levels 3 and 5 weeks after transplantation of CP-MSCs (*P* < 0.05; Figure 3(c)). These results were confirmed by immunofluorescence staining (Figure 3(d)). MiR-33 represses its target genes, which are involved in free fatty acid oxidation, such as CPT1A [23]. To evaluate whether miR-33 is a posttranscriptional regulator of CPT1A in BDL rat liver, we analyzed the expression levels of miR-33. As expected, we determined that miR-33 expression was reduced in BDL rats and was restored by transplantation of CP-MSCs (Figure 3(e)). Taken together, these results suggest that CPT1A may be regulated posttranscriptionally by miR-33 in a PPAR*α*-independent manner.

### 3.4. CP-MSC Transplantation Restores Cellular ATP Production by Regulating Heme Oxygenases

**Figure 4:**
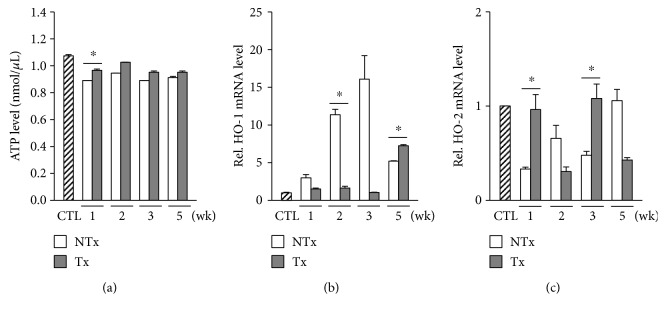
Changes in cellular ATP production and expression levels of HOs after BDL and/or CP-MSC transplantation. (a) Analysis of ATP levels in the liver tissues by the ATP assay. mRNA expression levels of HO-1 (b) and HO-2 (c), assessed by real-time PCR. GAPDH was used as an internal control for normalization. Data are expressed as a fold change related to the control group. ^∗^*P* < 0.05 (compared to the nontransplanted group). CTL: control group; NTx: nontransplanted group; Tx: transplanted group.

To demonstrate alterations in cellular energy production after BDL, we measured the ATP levels in BDL rat liver. ATP production was decreased after BDL but was augmented 1 week after CP-MSC transplantation (Figure 4(a)). Heme oxygenases (HOs) are suggested to be involved in regulating mitochondrial function [24]. Therefore, we assessed the expression levels of HOs in liver tissues. We determined that HO-1 expression was increased substantially in a time-dependent manner post-BDL until week 3. However, the augmented expression of HO-1 reverted to near-control levels 2 weeks after transplantation of CP-MSCs (*P* < 0.05; Figure 4(b)). The HO-2 expression pattern was inversely related to that of HO-1 (Figure 4(c)). These findings implicate that CP-MSC transplantation may ameliorate cellular ATP production via alternative expressions of HO-1 and HO-2.

## 4. Discussion

In this study, we demonstrated that alterations in lipid metabolism in BDL rats might be ameliorated by transplantation of CP-MSCs. Chronic cholestasis, resulting from BDL, led to massive inflammation, hypercholesterolemia, and a drastic decrease in intracellular fatty acid transport; these changes were partially reverted by CP-MSC transplantation. Regarding mitochondrial *β*-oxidation, the expression of CPT1A was changed following BDL and CP-MSC transplantation via miR-33, which is known as a posttranscriptional regulator of CPT1A, independent of PPAR*α*. Decreased cellular ATP production after BDL, which reflects mitochondrial dysfunction, was increased by CP-MSC transplantation via regulation of HO-1 and HO-2.

Stem cell therapy with MSCs has been tried for the treatment of various liver diseases, including cirrhosis and hepatic failure, as an alternative to liver transplantation. We previously reported that CP-MSCs had anti-inflammatory, antifibrotic, and proregenerative effects in a chronic liver injury model induced by carbon tetrachloride (CCl_4_) [10, 11]. Liver fibrosis and increased expression of type I collagen and *α*-smooth muscle actin in CCl_4_-treated rats were reduced after CP-MSC transplantation, which suggested that CP-MSCs have antifibrotic effects [10]. Transplantation of CP-MSCs also showed anti-inflammatory effects of attenuating leukocyte infiltration and augmenting anti-inflammatory cytokine interleukin 10 in liver tissues. In addition, CP-MSC transplantation promoted liver regeneration through activating autophagy [11]. In our present study, we demonstrated a novel effect of CP-MSCs as modulators of hepatic lipid metabolism in a BDL rat model. Alterations in serum cholesterol profiles and hepatic fatty acid oxidation, which resulted from BDL, were ameliorated after CP-MSC transplantation.

Because bile acids play a key role in lipid and energy homeostasis, alterations in lipid metabolism are inevitable in cholestatic liver diseases [4, 5, 25]. De Vriese and colleagues reported the results of lipid analysis of BDL rats and identified hypercholesterolemia and changes in the serum phospholipid profile, in proportion to serum levels of total bilirubin and ALP; however, a decrease in liver fat content in BDL rats was also observed [4]. In a more recent study, a high-cholesterol diet was not found to cause hepatic steatosis in BDL mice [25]. Our study findings of hypercholesterolemia without hepatic steatosis in BDL rats are consistent with those of these previous studies. Also, we demonstrated that intracellular fatty acid transport was markedly suppressed after BDL. The absence of hepatic steatosis, despite hypercholesterolemia, might be explained by intestinal lipid malabsorption via bile acids combined with the suppression of fatty acid import into hepatocytes.

Because the previous studies, which reported the changes in lipid metabolism in cholestatic liver diseases, focused on lipid malabsorption and cholesterol profiles, alterations in fatty acid oxidation have not been elucidated so far. Mitochondrial *β*-oxidation is a catabolic process that yields acetyl-CoA from long-chain acyl-CoA; acetyl-CoA then serves as a substrate in ATP generation [26]. Fatty acids, in the form of acyl-CoA, enter mitochondria by CPT1A, a rate-limiting enzyme that catalyzes mitochondrial *β*-oxidation [21]. Moreover, PPAR*α* has been identified as an upstream regulator of CPT1A [22]. We demonstrated that protein expression of CPT1A was upregulated in BDL rats and was downregulated after CP-MSC transplantation, independent of PPAR*α*. In contrast, mRNA expression of CPT1A exhibited an opposite pattern to CPT1A protein expression. Therefore, we explored the possibility of posttranscriptional regulation of CPT1A and verified that CPT1A is changed via alternative expression of miR-33 [23]. Because mitochondrial *β*-oxidation is a major source of ATP production in the liver [26], we further analyzed ATP production as an estimation of mitochondrial function. We revealed that decreased ATP production in BDL rat liver was restored by transplantation of CP-MSCs. HOs are thought to be mediators by which CP-MSCs correct mitochondrial dysfunction. Although HO-1 has been suggested to play a role in regulating mitochondrial function [24, 27], further studies are warranted to ascertain whether mitochondrial fatty acid oxidation is regulated by HOs. We have failed to demonstrate a consistent therapeutic effect on ATP production over time after CP-MSC transplantation. It may be worthwhile to transplant CP-MSCs repeatedly to overcome these limitations and to augment the therapeutic effect.

## 5. Conclusions

In our present study, we delineated perturbed lipid homeostasis in a model of chronic cholestatic liver injury. We demonstrated the therapeutic effect of CP-MSC transplantation to ameliorate alterations in lipid metabolism involving mitochondrial fatty acid oxidation. These results provide a novel insight into the mechanisms of stem cell therapy and support the therapeutic potential of CP-MSC transplantation in chronic cholestatic liver diseases.

## Supplementary Material

SUPPLEMENTARY FIGURE 1: The results of blood chemistry. P < 0.05 (compared to nontransplanted group). CTL, control group; NTx, nontransplanted group; Tx, transplanted group. SUPPLEMENTARY FIGURE 2: mRNA expression levels of ACSLs. β-actin was used as internal control for normalization. Data are expressed as a fold change related to the control group. ∗ P < 0.05 (compared to nontransplanted group). CTL, control group; NTx, nontransplanted group; Tx, transplanted group. SUPPLEMENTARY FIGURE 3: mRNA expression levels of FATPs. β-actin was used as internal control for normalization. Data are expressed as a fold change related to the control group. ∗ P < 0.05 (compared to nontransplanted group). CTL, control group; NTx, nontransplanted group; Tx, transplanted group.

## Figures and Tables

**Table 1:** Primer sequences.

Gene		Sequence
*ACSL 1*	Forward	5′-AAG CTC TGG AGG ATC TTG GA-3′
	Reverse	5′-GGG TTG CCT GTA GTT CCA CT-3′
*ACSL 3*	Forward	5′-TAA AGG CTG ACG TGG ACA AG-3′
	Reverse	5′-CCT TTG GAA TTC CTG TGG AT-3′
*ACSL 4*	Forward	5′-ATC TCC CAA AGC TGG AAC AC-3′
	Reverse	5′-CTG GTC CCT TAA CGT GTG TG-3′
*ACSL 5*	Forward	5′-TGT AGG GAT TGA GGG AGG AG-3′
	Reverse	5′-CAC AGC AAG TCC TCT TTG GA-3′
*FATP 1*	Forward	5′-CCC TGG ATG AGA GAG TCC AT-3′
	Reverse	5′-GCA GGA GAA ACA CCT GAA CA-3′
*FATP 2*	Forward	5′-CTC TTT CAG CAC ATC TCG GA-3′
	Reverse	5′-CCT CTT CCA TCA GGG TCA CT-3′
*FATP 3*	Forward	5′-CTG GGA CGA GCT AGA GGA AG-3′
	Reverse	5′-GCT GAG GCC AGA GGT CTA AC-3′
*FATP 4*	Forward	5′-CGC TGC TGT TCT CCA AGC TGG-3′
	Reverse	5′-GAT GAA GAC CCG GAT GAA ACG-3′
*FATP 5*	Forward	5′-GAA GGA ACC TGG AAG CTC TG-3′
	Reverse	5′-AGT GTC GAT TTC CGA TTT CC-3′
*FATP 6*	Forward	5′-CAG TAC CAC CAA GCC ATC AC-3′
	Reverse	5′-TGG AAC TGG CTA ATC ACA GC-3′
*PPARα*	Forward	5′-AGC CAT TCT GCG ACA TCA-3′
	Reverse	5′-CGT CTG ACT CGG TCT TCT TG-3′
*CPT1A*	Forward	5′-GCT TCC CCT TAC TGG TTC C-3′
	Reverse	5′-AAC TGG CAG GCA ATG AGA CT-3′
*HO-1*	Forward	5′-TGC ACA TCC GTG CAG AGA AT-3′
	Reverse	5′-CTG GGT TCT GCT TGT TTC GC-3′
*HO-2*	Forward	5′-AGG GCA GCA CAA ACA ACT CA-3′
	Reverse	5′-TCT GGC TCA TTC TGT CCT AC-3′
*β-Actin*	Forward	5′-GGG ACC TGA CTG ACT ACC TCA T-3′
	Reverse	5′-ACG TAG CAC AGC TTC TCC TTA AT-3′
*Gapdh*	Forward	5′-TCC CTC AAG ATT GTC AGC AA-3′
	Reverse	5′-AGA TCC ACA ACG GAT ACA TT-3′

## Abbreviations

ACSL: Long-chain fatty acyl-CoA synthetase
ALP: Alkaline phosphatase
ATP: Adenosine triphosphate
BDL: Bile duct ligation
BSA: Bovine serum albumin
CCl4: Carbon tetrachloride
CP-MSCs: Chorionic plate-derived MSCs
CPT1A: Carnitine palmitoyltransferase 1A
CRP: C-reactive protein
DAPI: 4′,6-Diamidino-2-phenylindole
DMEM/F12: Dulbecco's modified Eagle medium/Ham's F-12 medium
ELISA: Enzyme-linked immunosorbant assay
FATP: Fatty acid transport protein
FBS: Fetal bovine serum
HDL: High-density lipoprotein
hFGF-4: Human fibroblast growth factor-4
HO: Heme oxygenase
HRP: Horseradish peroxidase
LDL: Low-density lipoprotein
miR: MicroRNA
MSCs: Mesenchymal stem cells
PBS: Phosphate-buffered saline
PCR: Polymerase chain reaction
PD-MSCs: Placenta-derived MSCs
PPAR*α*: Peroxisome proliferator-activated receptor *α*
SDS-PAGE: Sodium dodecyl sulfate polyacrylamide gel electrophoresis
TBS: Tris-buffered saline.
