# Supplementary material for: Human Chorionic Plate-Derived Mesenchymal Stem Cells Restore Hepatic Lipid Metabolism in a Rat Model of Bile Duct Ligation
Source: Stem Cells Int. 2017 Nov 9;2017:5180579. doi: 10.1155/2017/5180579 (PMC5700509; doi:10.1155/2017/5180579)
Supplement: Supplementary file 1 — SUPPLEMENTARY FIGURE 1: The results of blood chemistry. P < 0.05 (compared to nontransplanted group). CTL, control group; NTx, nontransplanted group; Tx, transplanted group. SUPPLEMENTARY FIGURE 2: mRNA expression levels of ACSLs. β-actin was used as internal control for normalization. Data are expressed as a fold change related to the control group. ∗ P < 0.05 (compared to nontransplanted group). CTL, control group; NTx, nontransplanted group; Tx, transplanted group. SUPPLEMENTARY FIGURE 3: mRNA expression levels of FATPs. β-actin was used as internal control for normalization. Data are expressed as a fold change related to the control group. ∗ P < 0.05 (compared to nontransplanted group). CTL, control group; NTx, nontransplanted group; Tx, transplanted group. [file 5180579.f1.docx]

**Human Chorionic Plate-Derived Mesenchymal Stem Cells Restore Hepatic Lipid Metabolism in a Rat Model with Bile Duct Ligation**

**Online-only Supplements**

**Contents**

| Supplementary Figure 1. The results of blood chemistry. | p. 2 |
| --- | --- |
| Supplementary Figure 2. mRNA expression levels of ACSLs. | p. 3 |
| Supplementary Figure 3. mRNA expression levels of FATPs. | p. 4 |





Supplementary Figure 1: The results of blood chemistry. ^*^ *P* < 0.05 (compared to nontransplanted group). CTL, control group; NTx, nontransplanted group; Tx, transplanted group.





Supplementary Figure 2: mRNA expression levels of ACSLs. β-actin was used as internal control for normalization. Data are expressed as a fold change related to the control group. ^*^ *P* < 0.05 (compared to nontransplanted group). CTL, control group; NTx, nontransplanted group; Tx, transplanted group.





Supplementary Figure 3: mRNA expression levels of FATPs. β-actin was used as internal control for normalization. Data are expressed as a fold change related to the control group. ^*^ *P* < 0.05 (compared to nontransplanted group). CTL, control group; NTx, nontransplanted group; Tx, transplanted group.
